# Autonomous defect estimation in aluminum plate and prognosis through stochastic process modeling

**DOI:** 10.1038/s41598-025-13189-8

**Published:** 2025-08-12

**Authors:** Mrudul Jambulkar, Shivam Ojha, Amit Shelke, Anowarul Habib

**Affiliations:** 1 Department of Electrical Engineering, Indian Institute of Technology Bombay, Powai, Mumbai, Maharashtra 400076 India; 2 Department of Civil Engineering, Indian Institute of Technology Guwahati, Guwahati, Assam 781039 India; 3 Department of Physics and Technology, UiT The Arctic University of Norway, 9037 Tromsø, Norway

**Keywords:** Acoustic imaging, Scanning acoustic microscopy, Structural health monitoring, Short time fourier transform, Engineering, Aerospace engineering, Metals and alloys

## Abstract

The structural integrity and longevity of aluminum alloy components in lightweight engineering require accurate and efficient damage detection and prognosis methods. Traditional supervised machine learning (ML) techniques often face limitations due to dependency on large datasets, risk of overfitting, and high computational costs. To overcome these challenges, this study proposes an unsupervised learning framework that combines k-means clustering with a multi-phase gamma process to detect and model damage in aluminum plates. Scanning Acoustic Microscopy (SAM) images serve as the data source, from which comprehensive features are extracted in time, frequency, and time-frequency domains using Short-Time Fourier Transform (STFT). The K-means algorithm enables precise localization and sizing of surface defects without prior labels, while the gamma process captures the stochastic progression of damage over time. The method demonstrates high accuracy in estimating defect geometry and prognostic trajectories while maintaining low computational complexity. This unified, interpretable approach is generalizable to a range of materials and holds strong potential for real-time structural health monitoring (SHM) in safety-critical domains.

## Introduction

Damage detection has gained substantial attention in recent years due to its critical role in fields such as Structural Health Monitoring (SHM), non-destructive testing, and prognostic health management of engineering systems^1^. SHM is often integrated with damage prognosis, where the future state of a system is predicted through simulations based on prior knowledge and real-time data. The ability to predict damage progression is crucial, especially in mechanical and civil structures, to ensure safety and minimize economic losses associated with structural defects^2^. Lightweight structures are extensively used in industries such as aerospace, civil engineering, and automotive manufacturing. Lightweight design strategies aim to reduce the structural weight while preserving durability, thereby increasing productivity and optimizing resource utilization^3,4^. These structures provide significant environmental and structural benefits^5^. Among the materials used, aluminium and its alloys are particularly favored due to their high strength-to-weight ratio, ease of fabrication, workability, ductility, thermal conductivity, corrosion resistance, and aesthetically pleasing appearance^6^.

Despite these advantages, engineering structures are susceptible to damage from various adverse conditions. Early detection of such defects is essential for maintaining functionality and preventing failures^7^. Structural failures can occur due to poor design, faulty construction, foundation issues, excessive loads, and other factors. For instance, foundation failures may cause uneven stress distributions, leading to collapse. At the same time, extraordinary loads, such as those from heavy snowfalls, floods, earthquakes, and hurricanes, can severely compromise structural integrity^8^. Several techniques have been developed to detect and monitor damage in aluminum structures. Model-based methods use statistical processing and guided waves to locate defects. PL-SLDV phased arrays provide high-resolution, non-contact visualization using laser-induced vibrations^9^. Optical correlation techniques track surface strain to identify fatigue-related damage^10^. Acoustic emission methods detect real-time damage in hybrid aluminum/glass laminates^11^. Laser ultrasonic techniques combined with deep learning and energy mapping enhance detection performance. Elastic wave modulation spectroscopy detects microcracks through nonlinear wave interactions^12^. Together, these approaches form a comprehensive toolkit for structural health monitoring of aluminum components. To address similar challenges in metals, composites, and layered structures, additional methods have been developed. These include traditional techniques^13–15^, machine learning approaches^16–22^, and statistical signal analysis methods^23–27^. The core principle behind all these techniques is that structural damage alters physical properties such as stiffness, crack width, damping, or mass^1,27^. For instance, vibration-based SHM systems extract damage-related features from sensor-acquired signals to assess structural integrity^28–33^. In such systems, the identification of the structural properties is achieved by analyzing the measured vibrations^34^. Although natural frequency-based methods have been widely used, they often fall short in precisely locating and quantifying the damage^35–38^.

Damage detection methods compare structural responses between damaged and undamaged states. Various characteristics are measured and subsequently analyzed using signal processing techniques^39,40^ or machine learning algorithms^41,42^. Recently, Artificial Intelligence (AI)-driven approaches have gained prominence, offering solutions to complex problems when sufficiently high-quality datasets are available. However, the performance of these AI models heavily depends on the quality of the training data^34^. Signal processing remains a pivotal element in damage detection frameworks, as it enables the extraction of subtle changes in structural responses. Feature extraction techniques across time, frequency, and time-frequency domains have been extensively utilized. While time-domain analysis provides direct qualitative insights, it lacks clear frequency information. Frequency-domain analysis, enabled by the Fast Fourier Transform (FFT), reveals spectral content but cannot capture how these characteristics evolve over time. Consequently, time-frequency analysis methods, such as the Short-Time Fourier Transform (STFT), are employed to analyze signals in both domains simultaneously. The STFT partitions a signal into smaller time windows, computing the Fourier Transform of each segment to observe temporal variations in frequency content. However, the method’s accuracy is influenced by the size of the time window, with the uncertainty principle stating that achieving both high time and frequency resolutions simultaneously is not possible^43^.

Further, damage prognosis aims to forecast the future condition of a system based on its current damaged state, historical data, and anticipated operational environments. This capability is particularly vital for civil infrastructures vulnerable to degradation from events such as earthquakes, where accurate forecasting can save lives, accelerate post-event recovery, and reduce economic losses^2^. Numerous data-driven methods, including deep learning, recurrent neural networks (RNNs), and meta-learning approaches, have been explored for Remaining Useful Life (RUL) prediction^44^. Long Short-Term Memory (LSTM) networks have been employed to estimate RUL and model performance degradation in engines^45,46^. More recently, architectures like the Multiple Convolutional Long Short-Term Memory (MCLSTM) have improved RUL prediction accuracy by capturing temporal-spatial correlations in degradation signals^47^. Nevertheless, data-driven approaches are often constrained by low interpretability and the necessity of extensive training datasets. Particle filter-based prognostics have also been proposed^48^, although they can be computationally intensive and less accurate in high-dimensional spaces. Existing research predominantly addresses damage identification, with relatively fewer studies focusing on integrated damage prognosis. Many approaches treat damage identification and prognosis separately, highlighting the need for unified frameworks that integrate both tasks seamlessly.

This study proposes an approach that extracts features from the time, frequency, and time-frequency domains to enhance defect estimation. STFT is specifically utilized for time-frequency analysis to capture important transient signal characteristics. Incorporating all three domains ensures comprehensive feature representation, minimizing the risk of overlooking critical information. K-Means clustering is employed to estimate defect sizes by distinguishing anomalous from normal signal patterns. By partitioning signals into two clusters, the method efficiently identifies anomalies associated with structural defects. Defect diameters serve as a damage index, which is then modeled using a gamma process for prognosis. Unlike supervised deep learning models, this unsupervised technique does not require labeled training data, making it highly suitable for scenarios characterized by sparse or low-fidelity datasets. The approach offers low computational complexity, prevents overfitting, and provides greater interpretability, especially considering that damage in civil structures typically follows a gamma distribution. To validate the proposed methodology, damaged aluminum plates are examined using Scanning Acoustic Microscopy (SAM). SAM is a powerful label-free imaging technique widely employed in biomedical imaging, non-destructive testing, and material research^49–51^. It enables precise visualization of surface and subsurface features, offering both qualitative inspection and quantitative analysis capabilities crucial for structural diagnostics.

## Experimental procedure

### Sample preparation

An aluminum sheet of grade 7075 was selected for the experiments due to its high strength and favorable mechanical properties. Zinc is the primary alloying element in 7075 aluminum, which contributes to its excellent fatigue strength and superior corrosion resistance, particularly when compared to 2000-series aluminum alloys. Its strength is comparable to many steel grades, making it ideal for applications that require a combination of low weight and high strength. Common uses include aerospace structures, automotive components, medical devices, bicycle parts, and rock-climbing equipment. Although it is more expensive than other aluminum grades, its unique properties justify its use in demanding applications where performance is critical. In less critical scenarios, more cost-effective alloys may be suitable alternatives. For the purpose of high-resolution acoustic imaging, the aluminum sheet was sectioned into smaller samples to facilitate localized inspection using SAM. To simulate damage conditions, a high-speed Dremel drill was employed, using various drill bits to introduce controlled defects into the samples. The alloy’s medium machinability in the annealed condition allowed for the creation of precise and repeatable damage patterns, suitable for evaluation under SAM.

### Scanning acoustic microscopic imaging

**Fig. 1:**
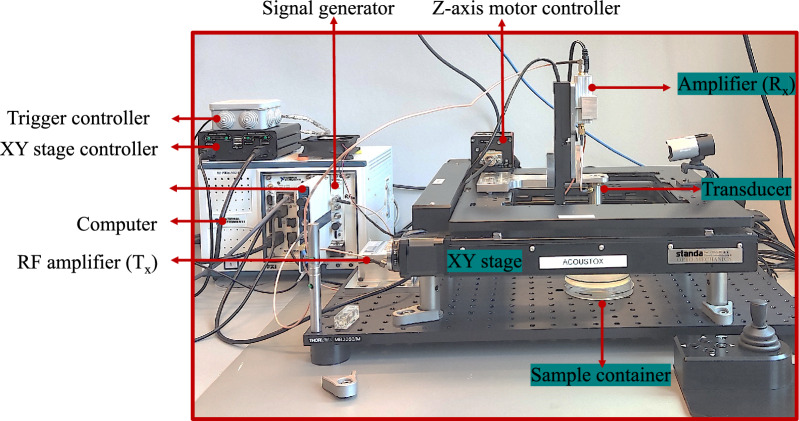
Representation of the SAM setup, which includes a signal generator, RF amplifier ($T_x$), and signal receiver amplifier ($R_x$) to excite and detect ultrasonic signals. The system is integrated with an XY stage and Z-axis motor controller for precise spatial positioning of the transducer. Automated scanning and data acquisition are managed by a computer-based control unit linked to the trigger and stage controllers. The sample is positioned in a dedicated container to allow controlled ultrasonic wave propagation^55–57^.

Figure 1 provides a detailed representation of the configured SAM setup, illustrating the system used for imaging the aluminum samples. The figure includes detailed annotations. It highlights key components and operational configurations. These serve as a visual reference for understanding the SAM imaging process. SAM operates in both reflection and transmission modes, each providing unique insights into the material’s internal properties. These imaging modes also allow visualization of subsurface structures, enabling more detailed analysis of the sample. For further details on the underlying principles and imaging methodologies of SAM, the numerical and imaging aspects are thoroughly explored in^52–54^.

This work relies on the use of Scanning Acoustic Microscopy (SAM) in reflection mode for imaging. The system uses a concave sapphire lens rod. It concentrates the acoustic energy onto the sample. Water is used as the coupling medium. This offers effective transmission of acoustic waves. Ultrasound signals are generated by a signal generator and directed toward the sample. The reflected waves, resulting from interactions with the sample’s surface, are captured and transformed into digital signals, referred to as A-scans or amplitude scans. To generate a C-scan image, the process is repeated across various positions within the XY plane by moving the sample using an X–Y stage. This repeated scanning process collects information from many locations. The collected information is compiled into an accurate 3D image in the direction of X, Y, and Z. This provides valuable information on the internal structure and properties of the sample.

The imaging system employed a 40 MHz PVDF-focused Olympus transducer with specific features, including a 6.35 mm aperture and a 12.5 mm focal length. A custom-designed SAM, depicted in Fig. 1, was controlled using a LabVIEW program. This SAM was integrated with a Standa high-precision motorized XY scanning stage (8MTF-200-Motorized XY Microscope Stage) for data collection. Acoustic imaging was facilitated by National Instruments’ PXIe FPGA modules and FlexRIO hardware housed within a PXIe-1082 chassis. The setup employed an arbitrary waveform generator (AT-1212-NI) to excite the transducer using predefined Mexican hat wavelets, chosen for their sharp time localization and smooth frequency profile, enabling precise detection of material changes^52,53,58^. Once the transducer generated the excitation signal, the reflected acoustic waves from the material were collected and amplified using a high-frequency RF amplifier (AMP018032-T) to enhance signal strength and clarity. These amplified reflections were then digitized using a 12-bit high-speed digitizer (NI-5772) operating at a sampling rate of 1.6 gigasamples per second (GS/s). This high sampling rate ensured fine temporal resolution, allowing accurate capture of the ultrasonic waveform details needed for subsequent signal analysis.

## Development of the damage index

For an effective prognosis, a damage index that reflects the system’s condition is essential. This can be a direct or indirect measure of damage. Time-domain features, such as mean, RMS, and peak value, provide statistical insights into signal amplitude and energy distribution but may be insufficient for detecting complex structural changes. Frequency-domain features are obtained using the FFT. These features reveal important spectral characteristics. The fundamental frequency gives information about the stiffness of the material. Spectral entropy helps detect subtle or hidden changes in the signal. Power Spectral Density (PSD) shows which frequency ranges contain the most energy. Time-frequency features, derived from the Wavelet Transform, capture transient and non-stationary effects by analyzing signals at multiple scales. Integrating all three domains enhances damage detection accuracy, offering a robust framework for structural health monitoring. In this paper, defect size is evaluated using K-means clustering for prognosis, based on extracted time, frequency, and time-frequency domain features.

### K-means clustering

Most of the available data is arbitrary and is not labeled. Unsupervised learning algorithms like K-means clustering can be useful in such cases^59^. Clustering algorithms are used to partition the unlabeled data into clusters such that data objects within the same cluster have similar features but differ from those in other clusters. The K-means clustering algorithm works well if we can pre-identify the number of clusters. In this work, only two clusters will be used for defect estimation: one representing normal signals and the other representing defects. The K-means clustering algorithm is a partitional clustering algorithm that divides the data into clusters. The distance is calculated, and a data point is assigned to a cluster if the distance between the data point and the mean of the cluster is the closest. The standard K-means algorithm uses Euclidean distance as a metric^59^. Mathematically, given a dataset $X = \{x_1, x_2, \ldots , x_n\}$ of $d$-dimensional data points of size $n$,

1 $$\begin{aligned} X = \{x_1, x_2, \ldots , x_n\} \end{aligned}$$

is partitioned into $k$ clusters $C = \{c_1, c_2, \ldots , c_k\}$ such that

2 $$\begin{aligned} \bigcup _{i=1}^{k} c_i = X \quad \text {and} \quad c_i \cap c_j = \emptyset \quad \text {for} \quad i \ne j. \end{aligned}$$

The K-means algorithm aims to minimize the sum of squared errors for each $k$ cluster. That is,

3 $$\begin{aligned} \text {Minimize} \sum _{i=1}^{k} \sum _{x \in c_i} \Vert x - \mu _i\Vert ^2 \end{aligned}$$

where $\mu _i$ is the mean of the points in cluster $c_i$. The steps of the K-means algorithm are as follows:

1. Initialization: Select $k$ random centroids (centers of clusters).
2. Assignment: Assign each data point to the nearest centroid.
3. Recomputation: Recompute the centroids as the mean of all data points assigned to each centroid.
4. Reassignment: Reassign each data point to the new centroids based on the updated mean values.
5. Iteration: Repeat steps 3 and 4 until the cluster assignments no longer change.
6. Completion: Finish the process if there are no reassignments of data points to different centroids.

The data may require some preprocessing. In this work, the features are first normalized so that the algorithm works effectively. Standardizing all the features gives them equal weight, ensuring that redundant or noisy objects do not impact the performance of our model and that we have reliable data to work with. The K-means algorithm uses Euclidean distance as a distance metric, which can cause the algorithm to give inaccurate results if there are irregularities in the size of different features. Otherwise, some features will be given more importance than others. The K-means algorithm is applied with only two clusters. With such a small number, it is not significantly affected by the curse of dimensionality. to perform effectively in estimating defect locations and characteristics. This algorithm is scalable on large datasets and requires lower computational costs than various deep neural network architectures^59^.

## Stochastic process modelling

### Gamma process modelling

After estimating the defect diameters, the next step is to develop a surrogate model for structural damage. In this model, the defect diameter is used as a key indicator of damage, denoted as $\eta _{jt}$, where *j*th represents the specific location and *t* represents the time instant. This indicator provides a quantitative measure of damage severity at a specific location over time. To capture how the damage evolves, a stochastic process is used. This approach accounts for the inherent uncertainty and variability in the degradation behavior. Among the various stochastic processes available, the gamma process is particularly well-suited for this application. The gamma process is ideal for modeling monotonic, non-decreasing degradation processes, which are characteristic of many structural damage phenomena. It provides a robust framework for describing how damage accumulates over time in a way that reflects the natural progression of wear, stress, and environmental influences on the structure^60^. The gamma process is a continuous-time Markov process where the increments are independent and gamma-distributed^61^. For $0 \le s < t$, the distribution of the random variable $X(t) - X(s)$ is a gamma distribution $\Gamma (a(t-s), b)$^62^:

4 $$\begin{aligned} X(t) - X(s)&\sim \Gamma (a(t-s), b) \end{aligned}$$

5 $$\begin{aligned} f_{(a(t-s), b)}(x)&= \frac{b^{a(t-s)} x^{a(t-s)-1} e^{-b x}}{\Gamma (a(t-s))} \end{aligned}$$

Here, *a* is the shape parameter and *b* is the scale parameter. By the additivity property of the gamma distribution, *X*(*t*) also follows the distribution, $\Gamma (at,b)$^63^. Since the gamma process is monotonically increasing, it becomes a suitable choice for modeling damage that increases over time. In this study, we will assume that the damage indicator follows a gamma process. Let the shape and scale parameters in the undamaged state be $a_0$ and $b_0$, respectively. Suppose a shock at time instant $\tau + 1$ causes the scale parameter to change from $b_0$ to $b_1$, but the shape parameter $a_0$ remains constant. The increments in damage indicator can then be modeled as a two-phase gamma process^62^.

6 $$\begin{aligned} \Delta \eta _{jt}&= \eta _{j(t+t_0)} - \eta _{jt} \sim \Gamma (a_0 t_0, b_0), \quad t_0 < t \le \tau + 1 \end{aligned}$$

7 $$\begin{aligned} \Delta \eta _{jt}&= \eta _{j(t+t_0)} - \eta _{jt} \sim \Gamma (a_0 t_0, b_1), \quad \tau + 1 < t \le T \end{aligned}$$

Here, $\Delta \eta _{jt}$ is an increment in $\eta _{jt}$ from time *t* to $t + t_0$ where $t_0$ is the time difference between two consecutive measurements. The likelihood is given by the following equation^62^

8 $$\begin{aligned} L(b_1, \tau )&= \prod _{t=1}^\tau \left[ \frac{b_0^{a_0 t_0}}{\Gamma (a_0 t_0)} \Delta \eta _{jt}^{a_0 t_0 - 1} \exp (-\Delta \eta _{jt} b_0) \right] \nonumber \\&\quad \times \prod _{t=\tau +1}^{T-1} \left[ \frac{b_0^{a_0 t_0}}{\Gamma (a_0 t_0)} \Delta \eta _{jt}^{a_0 t_0 - 1} \exp (-\Delta \eta _{jt} b_1) \right] \nonumber \\&= \Gamma (a_0 t_0)^{-(T-1)} b_0^{\tau a_0 t_0} b_1^{(T-\tau ) a_0 t_0} \nonumber \\&\quad \times \prod _{t=1}^{T-1} \Delta \eta _{jt}^{a_0 t_0 - 1} \exp \left[ -\sum _{t=1}^\tau \Delta \eta _{jt} b_0 - \sum _{t=\tau +1}^{T-1} \Delta \eta _{jt} b_1 \right] \end{aligned}$$

Log-likelihood can be obtained by taking the logarithm of this function.

9 $$\begin{aligned} \mathcal {L}(b_1, \tau )&= \ln L(b_1, \tau ) \nonumber \\&= -(T-1) \ln \Gamma (a_0 t_0) + \tau a_0 t_0 \ln b_0 \nonumber \\&\quad + (T-\tau ) a_0 t_0 \ln b_1 + (a_0 t_0 - 1) \sum _{t=1}^{T-1} \ln \Delta \eta _{jt} \nonumber \\&\quad - \sum _{t=1}^\tau \Delta \eta _{jt} b_0 - \sum _{t=\tau +1}^{T-1} \Delta \eta _{jt} b_1. \end{aligned}$$

Now, to maximize the likelihood:

10 $$\begin{aligned} \frac{\partial L(b_1,\tau )}{\partial b_1} = -\frac{(T-\tau )a_0t_0}{b_1} + \sum _{t=\tau +1}^{T-1} \Delta \eta _{jt} = 0 \Rightarrow \hat{b_1} = \frac{(T-\tau )a_0t_0}{\sum _{t=\tau +1}^{T-1} \Delta \eta _{jt}} \end{aligned}$$

In this model, the parameter $b_1$ is a function of the change point $\tau$. To determine the optimal change point $\hat{\tau }$, the value of $\tau$ that maximizes the likelihood function must be identified. This corresponds to the time at which a change in the system is most likely to have occurred. The estimation of $\hat{\tau }$ and the corresponding parameter $b_1$ is carried out under the assumption that the parameters $a_0$ and $b_0$ are known. These parameters characterize the system’s behavior before the change and can be estimated from measurements taken when the system is in a healthy state, as described in^62^.

### Parameter update and prognosis

Let the changes in damage indicator ($\Delta \eta _ {jt}$) follow a gamma distribution with *a* and *b* as the shape and scale parameters, respectively. Suppose the conjugate prior of *b* follows a gamma distribution with shape and scale parameters as $\alpha _0$ and $\beta _0$ respectively. Then the prior, likelihood, and posterior will be given by the following equations:

Prior :

11 $$\begin{aligned} p(b; \alpha _0, \beta _0) = \frac{\beta _0^{\alpha _0 t_0} b^{\alpha _0 t_0 - 1} e^{-\beta _0 b}}{\Gamma (\alpha _0 t_0)} \end{aligned}$$

Likelihood:

12 $$\begin{aligned} p(\mathcal {D} \mid a, b)= & \prod _{t=1}^n \Gamma (\Delta \eta _{jt}; \mid at_0, b) = \prod _{t=1}^n \frac{b^{at_0} \Delta \eta _{jt}^{at_0-1} e^{-b \Delta \eta _{jt}}}{\Gamma (at_0)} \end{aligned}$$

13 $$\begin{aligned}= & \frac{b^{at_0} \Delta \eta _{j1}^{at_0-1} e^{-b \Delta \eta _{j1}}}{\Gamma (at_0)} \times \frac{b^{at_0} \Delta \eta _{j2}^{at_0-1} e^{-b \Delta \eta _{j2}}}{\Gamma (at_0)} \times \dots \times \frac{b^{at_0} \Delta \eta _{jn}^{at_0-1} e^{-b \Delta \eta _{jn}}}{\Gamma (at_0)} \quad \text {where } t_0 = t_i - t_{i-1} \, \forall \, i. \end{aligned}$$

Posterior:

14 $$\begin{aligned} & p(b \mid \mathcal {D}) \propto \frac{b^{a n t_0} e^{-b \sum _{i=1}^n \Delta \eta _{jt}} \prod _{t=1}^n \Delta \eta _{jt}^{a t_0 - 1}}{\Gamma (n a t_0)} \times \frac{\beta _0^{\alpha _0 t_0} b^{\alpha _0 t_0 - 1} e^{-\beta _0 b}}{\Gamma (\alpha _0 t_0)} \end{aligned}$$

15 $$\begin{aligned} & \quad p(b \mid \mathcal {D}) \propto \frac{b^{n a t_0 + \alpha _0 t_0 - 1} \beta _0^{\alpha _0 t_0} \prod _{t=1}^n \Delta \eta _{jt}^{a t_0 - 1} e^{-b \left( \sum _{i=1}^n \Delta \eta _{jt} + \beta _0 \right) }}{\Gamma (n a t_0) \Gamma (\alpha _0 t_0)} \end{aligned}$$

Since *b* has a conjugate prior, its posterior also follows a gamma distribution but with parameters - $\alpha _ 0 '$ and $\beta _0'$ given by^62^:

16 $$\begin{aligned} a_0'= & na + a_0; \quad \beta _0' = \beta _0 + \sum _{t=1}^n \Delta \eta _{jt} \end{aligned}$$

17 $$\begin{aligned} & p(b|\mathcal {D}) \propto \frac{\beta _0^{\alpha _0 t_0} b^{\alpha _0' t_0-1} \prod _{t=1}^n \Delta \eta _{jt}^{at_0-1} e^{-(\beta _0' b)}}{\Gamma (\alpha _0 t_0)\Gamma (nat_0)} \end{aligned}$$

The posterior distribution $p(b|\mathcal {D})$ gets continuously updated as new data becomes available; hence, the estimate of *b* also gets updated.

## Results and discussion

### Extracting signal features and developing the damage index

Several samples with different defect sizes were imaged using Scanning Acoustic Microscopy (SAM). Each sample measured 25 mm $\times$ 25 mm, and consisted of an aluminum plate containing a predefined defect. When acoustic waves interacted with the sample, they were reflected back and subsequently captured and converted into electrical signals for analysis. The analog signals are converted to discrete-time signals using a digitizer with a sampling frequency of 200 MHz. The transducer’s raster scanning motion was used on the sample surface. In the raster mode, the echo signals are collected at each plate point, and the signal information is recorded. The step size 0.05 mm was used in horizontal and vertical directions, thus acquiring $100 \times 100$ pixels. Thus, the acquired signals include those resulting from damage (defect) to the plate, which are classified as anomalous, while others represent normal conditions.

**Fig. 2:**
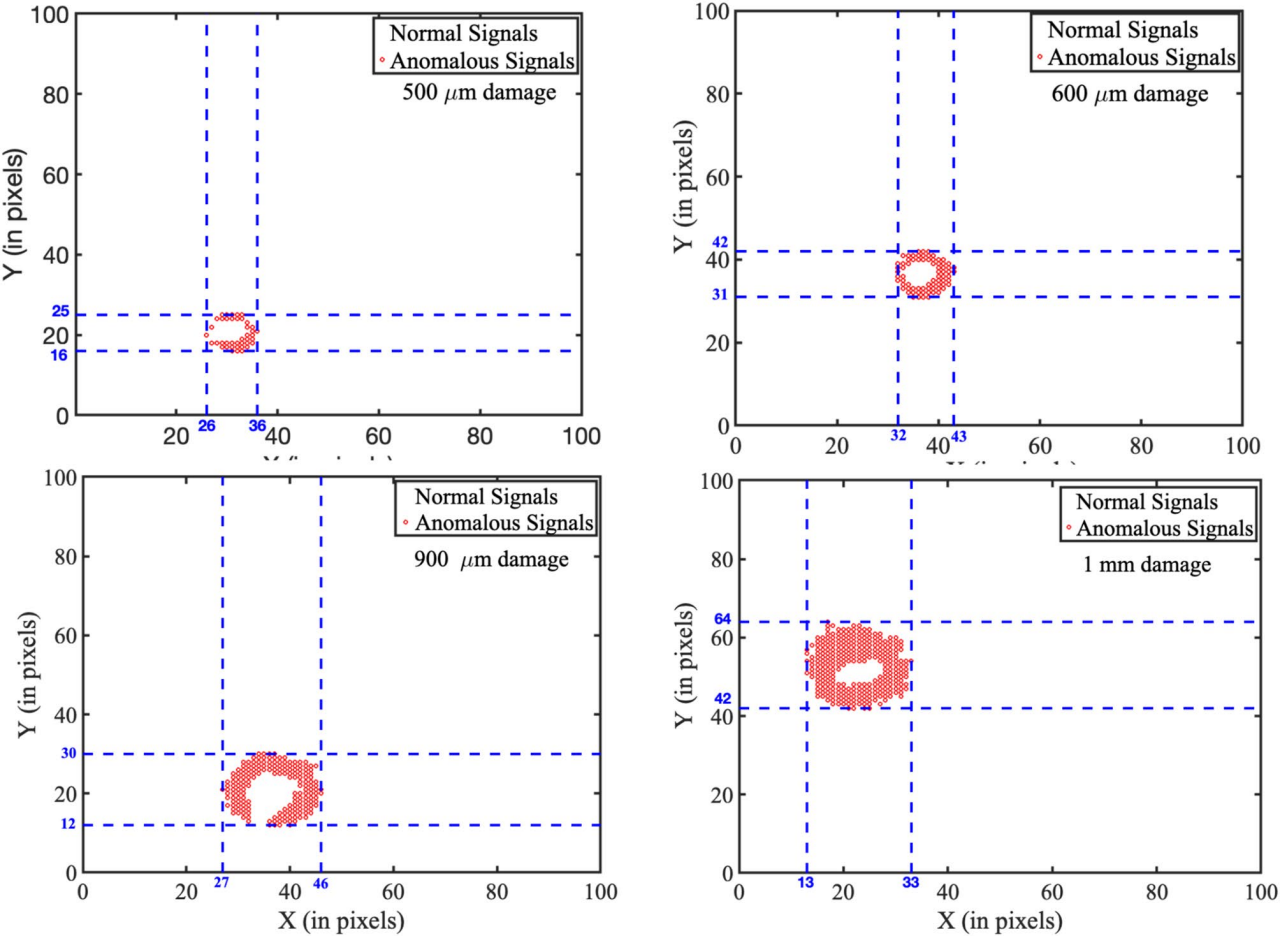
Clustering of anomalous and normal signals using K-means clustering, shown in four subfigures (**a**–**d**), representing different defect diameters as indicated in each subfigure. Subfigure (**a**) corresponds to a defect diameter of ($500 \upmu m$), showing a localized anomaly cluster (red circles) near the lower-left region. Subfigure (**b**) represents a defect diameter of ($600 \upmu m$), with a noticeable shift in the distribution of anomalous signals. Subfigure (**c**) depicts a defect diameter of ($900 \upmu m$), showing increased density and spread of anomalous signals across a broader region. Subfigure (**d**) corresponds to the largest defect diameter of (1*mm*), where anomalies consolidate into a distinct central cluster. The red circular regions represent cluster boundaries and centroids, illustrating the relationship between defect diameter and the spatial distribution of anomalous signals. Each coordinate in the graph corresponds to a signal at a specific spatial location. Anomalous signals (marked in red) indicate defect regions, while white areas denote normal signals from undamaged positions.

From these signals, features are extracted across the time domain (mean value, RMS value, peak value), the frequency domain (mean frequency, occupied bandwidth, power bandwidth), and the time-frequency domain (spectral skewness, time-frequency ridges). These extracted features are then normalized to acquire our training dataset. The K-means clustering algorithm was then employed to identify anomalous and normal signals based on these features. The experiment was conducted on $5.05 mm \times 5.05 mm$ aluminum plates with defect diameters of $500 \upmu m$, $600 \upmu m$, $900 \upmu m$, and $1000 \upmu m$. The results for these 4 samples are shown in Fig. 2. The cluster points are mapped onto their spatial locations, and the maximum pairwise distance among the anomalous cluster points is obtained to find the diameter of the defect. If any outliers were observed, they were ignored in this analysis. The estimated defect diameters are shown in Table 1. The results obtained are very close to the actual diameters.

**Table 1:** Comparison between the original diameters of the artificially created defects and their corresponding estimated values obtained through the proposed detection framework is presented. The results demonstrate that the framework can estimate defect dimensions with acceptable accuracy, supporting its applicability for quantitative damage assessment in aluminum plates.

Original diameter (${\upmu }m$)	Estimated diameter (${\upmu }m$)
500	510
600	625
900	1010
1000	1190

The estimation error for the largest defect (1 mm) reaches 19%, which is notably higher than for smaller defects. This can be attributed to several technical factors. In SAM, although high-resolution imaging is achievable, the lateral resolution is fundamentally limited by the acoustic wavelength and transducer properties. As the defect size increases, the acoustic signal tends to exhibit more diffuse boundary characteristics, making precise delineation difficult. This results in increased uncertainty during feature extraction and classification. In addition, K-means clustering introduces errors of approximation due to the assumption of spherical clusters with the same variance, which cannot hold for higher-order defects characterized by their irregular and spatially dispersed nature. In this case, signal variability and overlapping decision boundaries reduce the accuracy of estimation. As damage spreads over larger regions, the features may lose sensitivity to small spatial changes. This leads to a decline in classification performance.

In order to address these issues, several improvements are proposed. Applying advanced clustering algorithms such as Gaussian Mixture Models or Density-Based Spatial Clustering of Applications with Noise (DBSCAN) can better capture more complex spatial patterns^63,64^. Increasing the transducer frequency or incorporating signal deconvolution algorithms can provide higher spatial resolution and tighter borders. Additionally, incorporating spatial correlation features into the feature set offers a promising approach to improving discrimination between larger undamaged regions and long-damaged regions. Together, these advancements can significantly reduce estimation error, particularly for large or irregular defect morphologies.

### Surrogate modeling using gamma process

**Fig. 3:**
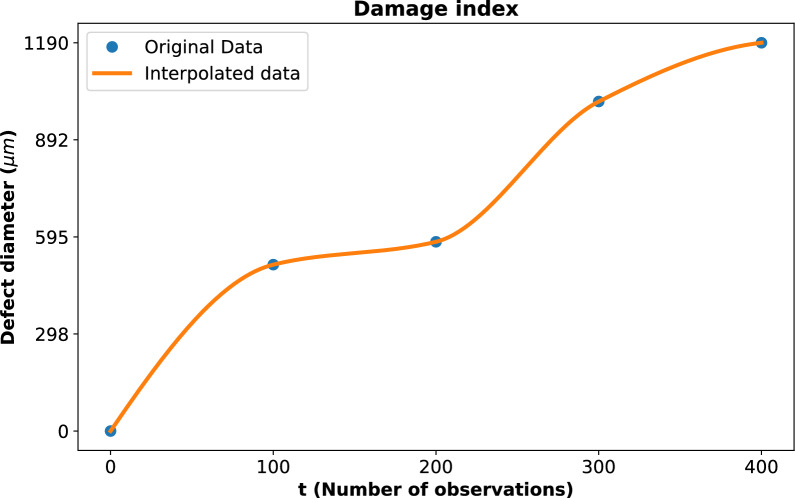
Damage index represented by defect diameter (${\upmu }m$) as a function of the number of observations. The blue dots indicate the original data points, while the orange line represents the interpolated data using PCHIP. The interpolation provides a continuous representation of the defect diameter progression.

Due to the limited number of data points in the damage index, interpolation was employed to estimate intermediate values. The Piecewise Cubic Hermite Interpolating Polynomial (PCHIP) method was used, as it preserves the shape and monotonicity of the data while providing smooth predictions between existing points. The technique provides a smooth and continuous curve of the damage development, as shown in Fig. 3. For the gamma process modeling, the initial shape parameter value, $a_0$, was assumed to be 6 and was kept constant throughout the analysis. Similarly, the initial rate parameter ($b_0$) was assigned a value of 1. Using these assumptions, the likelihood function was calculated to evaluate the fit of the gamma process to the given data.

The damage index is represented by defect diameter (${\upmu }m$) as a function of the number of observations, as shown in Fig. 3. The deep blue dots indicate the original data points obtained from experimental measurements, which represent discrete instances of observed damage progression. The orange line shows the interpolated data generated using PCHIP, a method chosen for its ability to preserve the monotonicity and shape of the data. The interpolation technique offers a smooth and continuous representation of defect diameter progression. It effectively bridges gaps between sparse data points and enables a more detailed understanding of damage evolution over time.

**Fig. 4:**
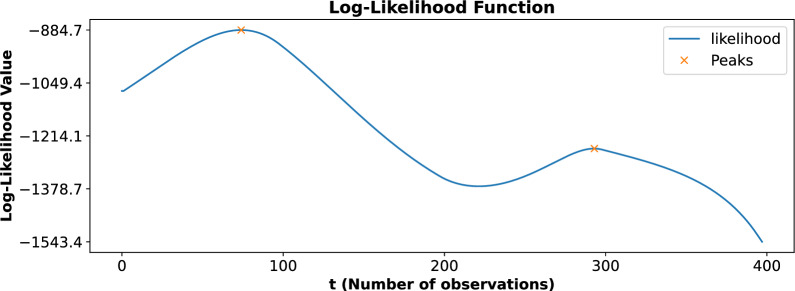
The likelihood function as a function of the number of observations (*t*). The blue curve represents the computed likelihood values, while the orange markers indicate the identified local maxima at time instants 74 and 293. These maxima correspond to critical change points in the damage progression, highlighting transitions in the structural degradation process and serving as the basis for the two-phase gamma process modeling.

Upon analyzing the complete dataset, the likelihood function revealed two prominent local maxima, as shown in Fig. 4. These peaks occurred at time instants 74 and 293, signifying critical points where the progression of damage exhibited noticeable changes. These time instants, referred to as change points, represent transitions in the behavior of the damage progression, indicating that the process is not uniform over time but instead evolves in distinct phases. To accurately capture these transitions and effectively model the damage progression, a two-phase gamma process modeling approach was employed. The first phase of the gamma process was applied to the time interval spanning from 0 to 293, with the first change point occurring at time instant 74. This phase represents the initial progression of damage, encompassing the behavior both before and after the first significant transition at time 74. The second phase of the gamma process was applied to the time interval from 74 to 400, with the second change point identified at time instant 293. This phase represents the progression of damage following the second major transition at time 293, indicating a distinct dynamic in the degradation process. By dividing the gamma process into two distinct phases, this modeling approach provided a more nuanced and accurate representation of the damage progression over time. The model effectively captured the evolving dynamics of the degradation process, with distinct transitions marked by identified change points. By differentiating between the pre- and post-transition phases, the two-phase modeling approach offers a clearer understanding of how damage accumulates over time. Detecting these behavioral shifts is crucial for enhancing the accuracy and reliability of structural health assessments.

The likelihood function, as illustrated in Fig. 4, represents the computed likelihood values as a function of the number of observations (*t*). The blue curve shows the variation of the likelihood values across the dataset, while the orange markers highlight two critical local maxima observed at time instants 74 and 293. These maxima are significant as they correspond to change points in the damage progression, indicating distinct transitions in the structural degradation process. The presence of these peaks in the likelihood function suggests that the damage evolution does not follow a uniform pattern but consists of two distinct phases separated by the change points. The first peak at $t=74$ marks the transition into the initial phase of damage progression, while the second peak at $t=293$ indicates the onset of a second degradation phase. These change points were instrumental in defining the two-phase gamma process model, which was employed to capture the temporal dynamics of the damage progression accurately. By identifying these critical points, the likelihood function provides a robust method for detecting transitions in structural behavior, enabling more detailed and precise modeling of the degradation process over time.

### Real-time updation of the gamma process for prognosis

**Fig. 5:**
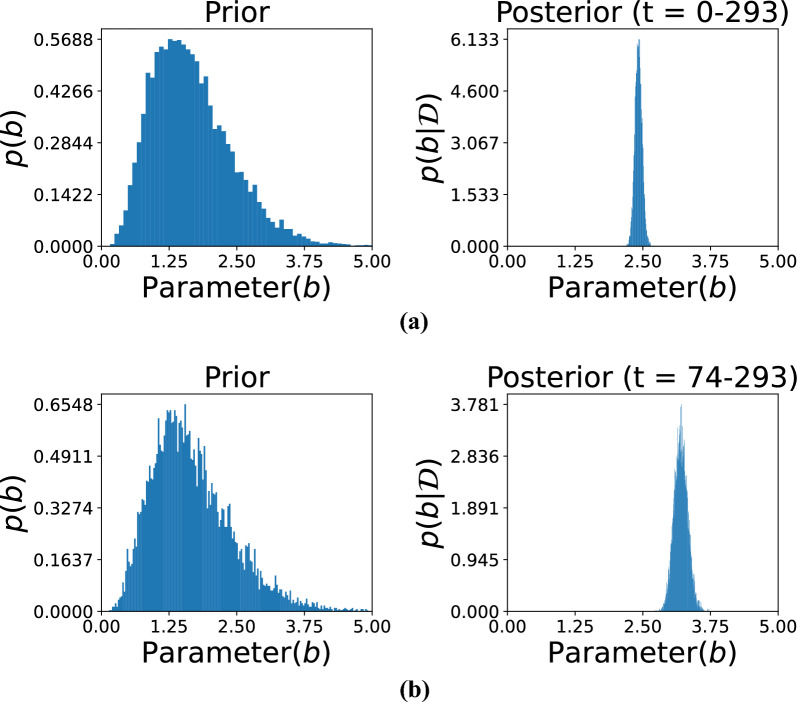
Comparison of the prior and posterior distributions of parameter *b* across different observation intervals. Subfigure (**a**) displays the distribution based on observations from 0 to 293, while subfigure (**b**) focuses on the updated distribution from observations 74 to 293. The comparison illustrates how incorporating new data refines the estimate of parameter *b* over time.

**Fig. 6:**
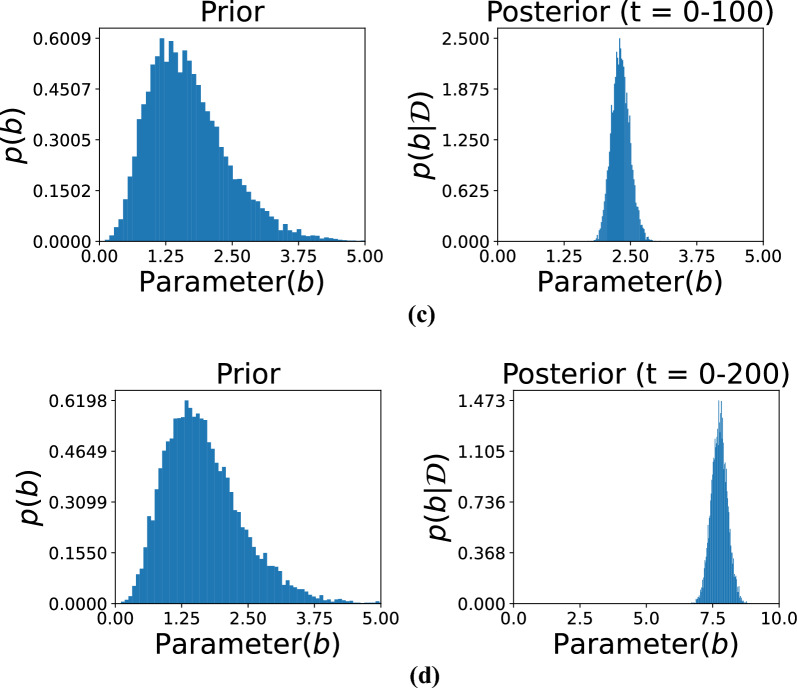
Posterior distributions of the model parameter *b* over large observation time spans. Subfigure (**c**) presents results based on time steps 0–100, whereas subfigure (**d**) depicts distributions based on time steps 0–200. These comparisons demonstrate how including more temporal information improves posterior estimation and thus enhances confidence in inferring parameters.

**Fig. 7:**
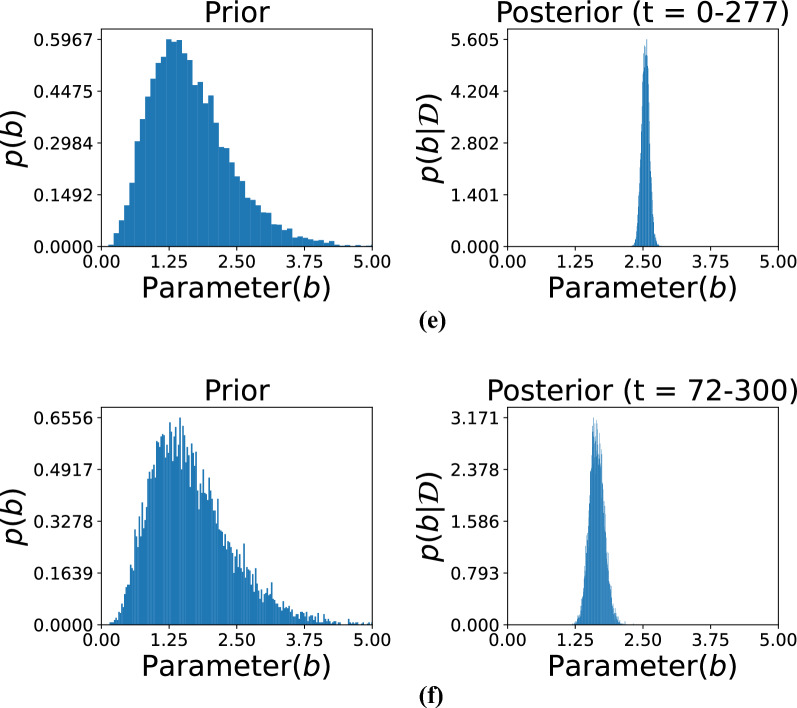
Visualization of the posterior distributions of the parameter evolving *b* from data covering different periods. Subfigure (**e**) presents the distribution according to early-period data (0–277), and subfigure (**f**) presents those from a later period (72–300). The contrast indicates how additional data focuses the estimate of *b*, monitoring dynamic change in system behavior over time.

Figures 5, 6, and 7 illustrate the comparison between prior and posterior distributions for the model parameter *b*, demonstrating the application of Bayesian inference in refining parameter estimates based on observed data across different time intervals. Each figure displays the prior and posterior distributions of $b$. The prior distribution reflects the initial assumptions, modeled as a gamma distribution with hyperparameters $\alpha _0 = 5$ and $\beta _0 = 3$. These parameters define the initial spread and central tendency of the distribution, establishing a prior baseline before incorporating any observed data. The posterior distribution of $b$, obtained by updating the prior with data from specific time intervals, is also presented. This allows for a refined estimation that reflects both prior knowledge and evidence from actual observations.

The subfigures in these three figures represent different temporal segments that capture distinct phases of the damage evolution process. Specifically, Fig. 5 includes subfigures (a) for time *t*= 0–293 and (b) for *t*= 74–293, Fig. 6 includes (c) for *t*= 0–100 and (**d**) for *t*= 0–200, and Fig. 7 includes (e) for *t*= 0–277 and (f) for *t*=72–300. These intervals were selected to reflect the evolving nature of structural degradation and allow an assessment of how time-dependent data influences the estimation of the parameter *b*.

Shorter observation windows, such as those in subfigures (c) and (d) of Fig. 6, often result in broader posterior distributions. This indicates higher uncertainty due to limited data, which constrains the model’s ability to infer the true parameter values with confidence. In contrast, longer intervals, such as those in Fig. 5, allow for the inclusion of more data points, which sharpens the posterior distribution and narrows the uncertainty. This trend illustrates the principle that increasing the volume of data over time leads to more accurate and confident estimates. Together, these figures highlight the importance of Bayesian inference in damage modeling, where prior knowledge is continually refined through the integration of new data. The shifting and narrowing of the posterior distributions visually and quantitatively emphasize the model’s increasing confidence in the parameter *b* as more evidence becomes available. Furthermore, the variation in posterior behavior across different time intervals underlines the significance of temporal data selection in predictive modeling. The ability of this approach to adaptively incorporate new information makes it particularly well-suited for structural health monitoring applications, where the degradation process is progressive and dynamic.

The hyperparameters $\alpha _0$ and $\beta _0$ for the prior distribution of *b* were set to 5 and 3, respectively. These hyperparameters determine the shape and scale of the prior distribution, thereby influencing its initial variance and central tendency. As shown in the figure, observational data from specific time ranges were then used to compute the posterior distributions. The subfigures correspond to the following time intervals: (a) *t*= 0–293, (b) *t*= 74–293, (c) *t*= 0–100, (d) *t*= 0–200, (e) *t*= 0–277, and (f) *t*= 72–300. Each time range reflects a different phase of the damage progression, providing insights into how parameter *b* evolves. The posterior distributions reveal the effect of the observed data in sharpening and shifting the initial assumptions represented by the priors. For instance, shorter time intervals, such as in subfigures (c) and (d), might yield less concentrated posterior distributions due to limited data, leading to broader uncertainty in the parameter estimates. Conversely, longer time intervals, as in subfigures (a) and (b), provide more precise posterior distributions with reduced uncertainty. This progression demonstrates how both the quantity and quality of data contribute to the refinement of parameter estimates. The figure illustrates the dynamic interaction between prior knowledge and observed data within the Bayesian framework. The prior distribution serves as the initial foundation for parameter estimation, while new observations iteratively update this foundation, leading to more accurate and confident results. Moreover, the selection of time intervals significantly influences the resulting posterior distributions, showing how the temporal resolution of data impacts the inference process. This approach is particularly effective for modeling time-dependent phenomena, such as damage progression in structures, where the parameter *b* captures critical characteristics of the underlying degradation behavior.

**Fig. 8:**
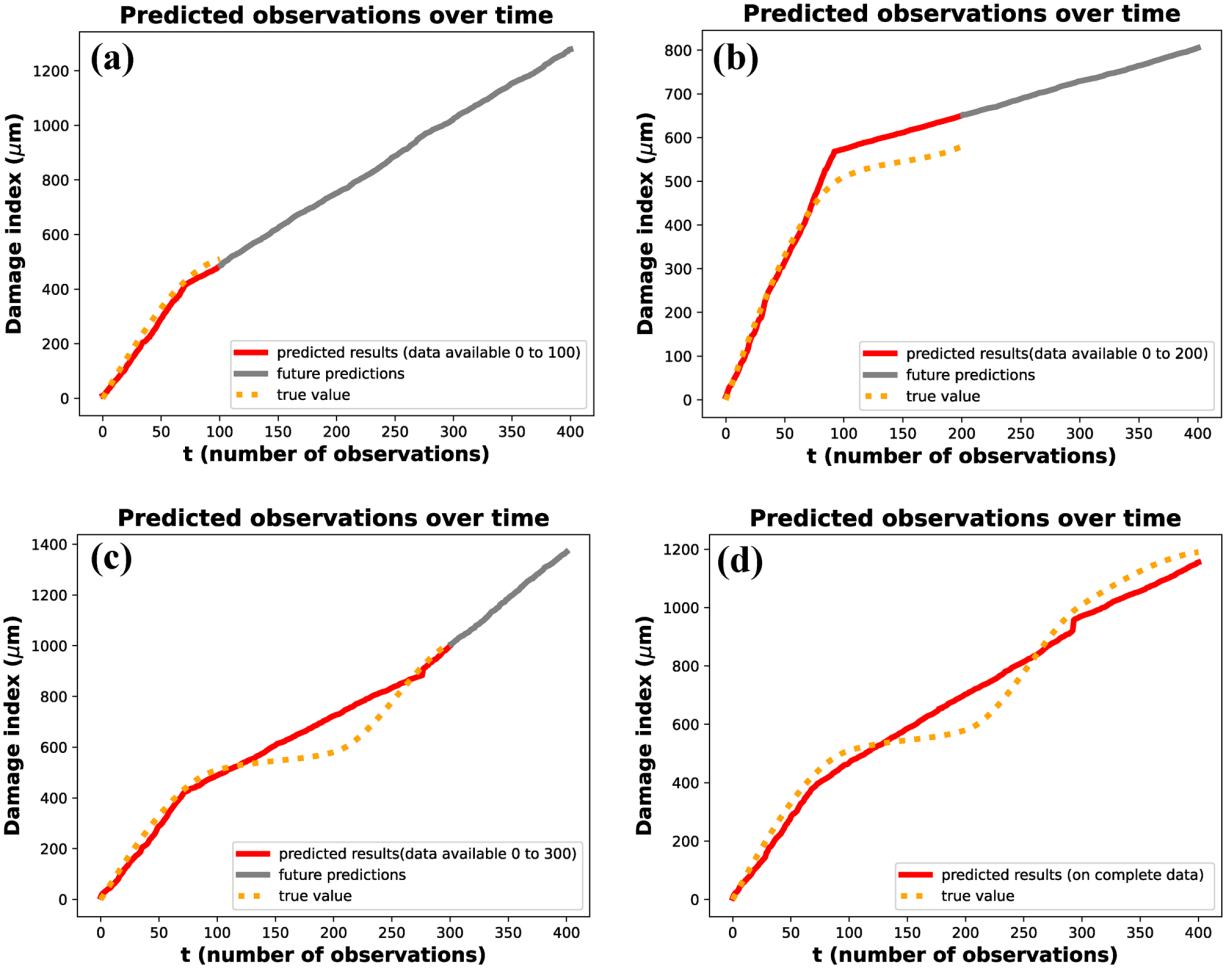
Predicted observations over time using gamma process modeling. The orange dotted curve represents the original data available up to a given time instant. The red curve illustrates the predicted results based on the available data, while the grey curve depicts the predicted future state of the system. Subfigures (**a**–**d**) show the predictions for data availability up to $t= 100$, $t= 200$, $t= 300$, and the complete dataset, respectively. The figure demonstrates the accuracy of predictions as the dataset increases in size.

The damage index progression over time was analyzed to evaluate the effect of available data on the accuracy of predictions, as illustrated in Fig. 8. The figure presents four scenarios where the predicted damage index is compared to the true values (${\upmu }m$) for varying levels of data availability: (a) data up to $t=100$, (b) data up to $t=200$, (c) data up to $t=300$, and (d) the complete dataset. In each subfigure, the red line represents the predicted results based on the available data, the orange dotted line indicates the future predictions, and the gray line shows the true values. The analysis demonstrates that as more data becomes available, the predictions become increasingly accurate and align more closely with the true damage progression. When the complete dataset was analyzed, two distinct change points were identified at $t=72$ and $t=277$. The estimated values of the parameter b from the posterior distributions were found to be 2.42 after the first change point and 3.20 after the second change point (Fig. 8c,d). This indicates that the damage progression exhibited two distinct phases, with a clear transition in the degradation dynamics after each change point. A two-phase gamma process was subsequently applied to model this behavior, effectively capturing the progression over time.

For partial datasets, different observations were made. When data from t = 0–100 was analyzed, a single change point was identified at $t= 72$, and the parameter *b* was estimated from the posterior distribution (Fig. 5). Similarly, when data from t = 0–200 was analyzed, a single change point was observed at $t=93$, with a corresponding parameter value of *b* obtained from the posterior distribution (Fig. 5f). However, in data from t = 0–300, two change points were identified at $t= 72$ and $t=277$, aligning with the results obtained from the complete dataset. These results highlight the impact of data availability on the ability to detect change points and estimate model parameters accurately. With smaller datasets, fewer change points were identified, and the predictions exhibited greater uncertainty, as reflected in the future predictions (orange dotted lines).

As additional data were incorporated, the posterior distributions became increasingly concentrated, resulting in improved estimates of parameter *b* and more accurate modeling of the damage progression. The value of *b* was computed as the mean of its posterior distribution. These estimated values were then used to model the system using a Gamma process, capturing the stochastic nature of cumulative damage over time. Figure 8 presents the predicted results and the predicted future state of the system based on this gamma process modeling at various time instants. Figure 8a–c represent the predicted and future damage index up to time instants 100, 200, and 300, respectively. Figure 8d shows the predicted results when complete data is available, closely following the true values. The application of a two-phase gamma process for prognosis provided a robust framework for capturing the non-linear progression of damage over time. This methodology demonstrates the importance of leveraging complete datasets whenever possible to improve the reliability and precision of damage modeling.

Thus, the damage index follows the following distribution on analyzing the complete data from 0–400 :

18 $$\begin{aligned} \Delta \eta _{jt}&= \eta _{j(t+1)} - \eta _{jt} \sim \Gamma (6 , 1), \quad 0 < t \le 73 \end{aligned}$$

19 $$\begin{aligned} \Delta \eta _{jt}&= \eta _{j(t+1)} - \eta _{jt} \sim \Gamma (6, 2.419), \quad 73 < t \le 292 \end{aligned}$$

20 $$\begin{aligned} \Delta \eta _{jt}&= \eta _{j(t+1)} - \eta _{jt} \sim \Gamma (6 , 3.203), \quad 292 < t \le 400 \end{aligned}$$

Figure 8 shows the predicted observations by this gamma process modeling across different time instants.

**Table 2:** Forecasting accuracy of the gamma process-based prognosis model evaluated at different partial observation horizons. RMSE and MAPE quantify the deviation between predicted and actual damage indices. Results demonstrate improved accuracy with increased observation time, validating the model’s adaptability to evolving damage progression.

Observation	RMSE	MAPE (%)
0–100	80.94	11.09
0–200	195.57	16.56
0–300	75.71	10.60
0–400	61.59	9.99

To quantitatively evaluate the accuracy of the damage prognosis model, a comparison between the predicted and actual damage indices was conducted using Root Mean Squared Error (RMSE) and Mean Absolute Percentage Error (MAPE). Table 2 presents the performance metrics across different partial observation intervals. For forecasts generated using data up to $t=100$, the RMSE was 80.94 and MAPE was 11.09%, indicating reasonable accuracy during early-stage degradation. When the observation window was extended to $t = 200$, the RMSE increased to 195.57 and the MAPE to 16.56%, reflecting a temporary drop in performance. This decline may be attributed to evolving damage dynamics that were not yet well captured by the model, possibly due to the use of interpolated data instead of original measurements and the inherent randomness of the gamma process. However, predictive accuracy improved markedly with further data: for $t=300$, the RMSE decreased to 75.71 and MAPE to 10.60%, while for *t*=400, the RMSE was 61.59 with a MAPE of 9.99%. These results demonstrate the model’s capacity to generate reliable forecasts with limited initial data, while progressively refining its accuracy as more observations become available. The inclusion of these quantitative metrics strengthens the validation of the proposed unsupervised framework for robust and data-efficient structural health prognosis.

The uncertainty in measurements, or the prediction interval, can be quantified by tracking the standard deviation as more data becomes available. Given that the increments of the damage index follow a Gamma process and are treated as independent random variables, the variance of the damage index at a specific time instant is equal to the cumulative sum of the variances of all previous increments. For a Gamma distribution with shape parameter *a* and scale parameter *b*, the variance of each increment is $a/b^2$. Therefore, the standard deviation of the damage index at time *t*, accounting for cumulative contributions, is given by^60,65,66^ :

$$\begin{aligned} \text {Standard Deviation}(\eta _{jt}) = \sqrt{\frac{a}{b^2} \cdot t} \end{aligned}$$

The figures show how the standard deviation changes with time. Let $\text {Standard Deviation}(\eta _{jt}) = \sigma (t)$

For data available 0–100 :

$$\begin{aligned} \sigma (t) = {\left\{ \begin{array}{ll} \sqrt{\dfrac{6}{1^2} \cdot t} = \sqrt{6t}, & 0< t \le 71 \\ \sqrt{\dfrac{6}{2.317^2} \cdot (t - 71)} + \sigma (71), & 71 < t \le 400 \end{array}\right. } \end{aligned}$$

For data available 0–200 :

$$\begin{aligned} \sigma (t) = {\left\{ \begin{array}{ll} \sqrt{\dfrac{6}{1^2} \cdot t} = \sqrt{6t}, & 0< t \le 92 \\ \sqrt{\dfrac{6}{7.748^2} \cdot (t - 92)} + \sigma (92), & 92 < t \le 400 \end{array}\right. } \end{aligned}$$

For data available 0–300 :

$$\begin{aligned} \sigma (t) = {\left\{ \begin{array}{ll} \sqrt{\dfrac{6}{1^2} \cdot t} = \sqrt{6t}, & 0< t \le 71 \\ \sqrt{\dfrac{6}{2.546^2} \cdot (t - 71)} + \sigma (71), & 71< t \le 276 \\ \sqrt{\dfrac{6}{1.646^2} \cdot (t - 276)} + \sigma (276), & 276 < t \le 400 \end{array}\right. } \end{aligned}$$

For data available 0–400 :

$$\begin{aligned} \sigma (t) = {\left\{ \begin{array}{ll} \sqrt{\dfrac{6}{1^2} \cdot t} = \sqrt{6t}, & 0< t \le 73 \\ \sqrt{\dfrac{6}{2.419^2} \cdot (t - 73)} + \sigma (73), & 73< t \le 292 \\ \sqrt{\dfrac{6}{3.203^2} \cdot (t - 292)} + \sigma (292), & 292 < t \le 400 \end{array}\right. } \end{aligned}$$

**Fig. 9:**
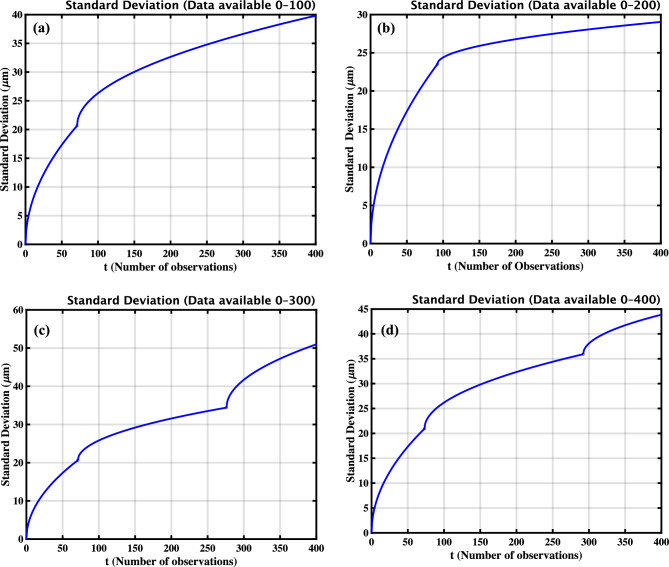
The standard deviation of the damage index is computed over varying amounts of observed data to assess signal stability and damage sensitivity in the PZT sample. Subfigures (**a**–**d**) display standard deviation curves calculated using increasing data lengths: (**a**) 100 observations, (**b**) 200 observations, (**c**) 300 observations, and (**d**) the complete dataset of 400 observations. Each plot illustrates the evolution of standard deviation over time, allowing for a comparison of forecasting uncertainty under different levels of data availability. As additional data are incorporated, the standard deviation increases, reflecting the cumulative uncertainty associated with the progression of the damage index.

To investigate the variability introduced by the stochastic nature of the damage evolution model, the standard deviation of the damage index was analyzed over time. Increments in the damage index are modeled using a Gamma process. As a result, the variance of the damage index increases over time. This is because the variance at any given time is the cumulative sum of the variances of all previous increments. This holds under the assumption that the increments are independent Gamma-distributed variables. Figure 9 illustrates this behaviour by showing standard deviation curves computed using increasing amounts of available data: 100, 200, 300, and 400 observations, corresponding to subfigures (a) through (d), respectively. As expected, the standard deviation of the damage index increases with the number of observations. This reflects the accumulating uncertainty associated with progressive damage over time. The analysis validates the suitability of the gamma process in capturing both the mean trend and the growing variability of the damage index, which is essential for accurate prognosis in structural health monitoring applications.

## Conclusion

This paper demonstrates the efficacy of a data-limited, unsupervised learning model that combines K-means clustering and a multi-stage gamma process for both defect detection and prognosis in aluminum structures. Clustering performances were successful in separating defective and healthy areas of the signal spatially for defects ranging from 500, 600, 900 $\upmu m$, and 1 mm, with identifiable localization patterns being observed as damage worsened. The PCHIP-interpolated damage index accurately represented the trend of development, while the likelihood function identified important damage evolution milestones at observation times *t* = 74 and *t* = 293. These served as the basis for initiating a two-phase Gamma process modeling. Prognostic forecasts generated using partial data (i.e., up to *t* = 100, *t* = 200, and *t* = 300) showed strong agreement with actual measurements, thereby validating the reliability of early predictions. The proposed methodology avoided excessive use of large labeled datasets with a high accuracy rate. It also ensured interpretability through the use of a stochastic gamma-based damage index that was derived based on natural degradation behavior. Through the use of comprehensive feature extraction in the time, frequency, and time-frequency domains through STFT, the model achieved resolution limitations that are typical of standard signal processing. Due to its low computational cost and broad applicability, the proposed approach is well-suited for real-time SHM applications in aluminum structures and other fields such as aerospace, civil, and mechanical systems, where early detection of small-scale damage is essential for ensuring safety and maintaining performance.

## Limitation and future direction

The proposed unsupervised framework effectively identifies and models single-stage deterioration in aluminum plates. However, it currently assumes piecewise-stationary damage progression. This limits its application to more general instances of multi-stage deterioration wherein damage behavior may exhibit sudden jumps, non-monotonicity, or shifts in underlying stochastic properties. Future work will extend the gamma process model to capture such multi-phase or hierarchical deterioration kinetics more realistically.

Enhancing predictive accuracy and interpretability can be achieved by integrating physical degradation models−such as fracture mechanics or fatigue crack growth laws−into the data-driven framework. Embedding such models within a Bayesian updating scheme would enable real-time updating of prognostic estimates while being consistent with known physics. The hybrid approach is particularly valuable in identifying damage at an early stage and in scenarios where data are sparse or uncertain. Future work will extend the framework to handle multiple damage sites and their interactions. This will make it more applicable to real-world structures. The method will also be adapted for complex materials, such as composites and anisotropic systems. In these materials, damage signals vary more due to directional properties and internal heterogeneity. From the signal analysis side, alternatives to the Short-Time Fourier Transform, such as wavelet transforms or learned signal embeddings, will be investigated to improve feature robustness against noise and low resolution. Experimental validation at a larger scale under varying operating and environmental conditions will also be required for real-world deployment. Incorporation of uncertainty quantification in both the clustering and prognosis stages will improve the reliability of the framework for real-time structural health monitoring applications.

## Data Availability

The data sets used and/or analyzed during the current study are available from the corresponding author upon reasonable request.
